# *Mycobacterium tuberculosis* Peptidyl-Prolyl Isomerases Also Exhibit Chaperone like Activity *In-Vitro* and *In-Vivo*

**DOI:** 10.1371/journal.pone.0150288

**Published:** 2016-03-16

**Authors:** Saurabh Pandey, Ashish Sharma, Deeksha Tripathi, Ashutosh Kumar, Mohd Khubaib, Manish Bhuwan, Tapan Kumar Chaudhuri, Seyed Ehtesham Hasnain, Nasreen Zafar Ehtesham

**Affiliations:** 1 Inflammation Biology and Cell Signaling Laboratory, National Institute of Pathology, New Delhi, India; 2 Dr. Reddy’s Institute of Life Sciences, University of Hyderabad Campus, Professor CR Rao Road, Hyderabad, India; 3 Molecular Infection and Functional Biology Laboratory, Kusuma School of Biological Sciences, Indian Institute of Technology, New Delhi, India; 4 Kusuma School of Biological Sciences, Indian Institute of Technology, New Delhi, India; Bose Institute, INDIA

## Abstract

Peptidyl-prolyl cis-trans isomerases (Ppiases), also known as cyclophilins, are ubiquitously expressed enzymes that assist in protein folding by isomerization of peptide bonds preceding prolyl residues. *Mycobacterium tuberculosis* (*M*.*tb*) is known to possess two Ppiases, PpiA and PpiB. However, our understanding about the biological significance of mycobacterial Ppiases with respect to their pleiotropic roles in responding to stress conditions inside the macrophages is restricted. This study describes chaperone-like activity of mycobacterial Ppiases. We show that recombinant rPpiA and rPpiB can bind to non-native proteins *in vitro* and can prevent their aggregation. Purified rPpiA and rPpiB exist in oligomeric form as evident from gel filtration chromatography.*E*. *coli* cells overexpressing PpiA and PpiB of *M*.*tb* could survive thermal stress as compared to plasmid vector control. HEK293T cells transiently expressing *M*.*tb* PpiA and PpiB proteins show increased survival as compared to control cells in response to oxidative stress and hypoxic conditions generated after treatment with H_2_O_2_ and CoCl_2_ thereby pointing to their likely role in adaption under host generated oxidative stress and conditions of hypoxia. The chaperone-like function of these *M*.*tuberculosis* cyclophilins may possibly function as a stress responder and consequently contribute to virulence.

## Introduction

The disease tuberculosis (TB) caused by *Mycobacterium tuberculosis* (*M*.*tb*), is the second largest killer after HIV-AIDS [1]. For successful colonization of human host, *M*.*tb* forms a niche by establishing molecular interaction networks within the host system. *M*.*tb* has evolved mechanisms to survive in macrophages that represent one of the most stressful environments for bacteria. Successful colonization of the intraphagosomal niche by the pathogen depends on molecular interaction network within the host [2,3]. The effector molecules which play crucial role in host pathogen interaction at the molecular level are mostly stress responders, HSPs, chaperones and other protein modifying enzymes [4]. The pathogen subverts host defenses by quenching ROS and RNS [5–7] disrupting the membrane repair [8], phagolysosomal fusion [9], suppression of autophagy [10] and by escaping immune challenges [11,12] and immune quorum sensing [13]. Bacterial chaperones play a vital role in protein folding and secretion, thereby indirectly contributing to the virulence and survival of the pathogen inside the host [14].

Prolyl isomerases, also known as cyclophilin, are expressed ubiquitously from bacteria to human and as of now 17 cyclophilin proteins in humans, 29 in *Arabidopsis* and 8 in *Saccharomyces* have been reported so far [15,16]. Isoform diversity, various subcellular localization and differences during evolution are indicative of their functional importance and acquisition of new roles. Prolyl isomerases lie in three structurally and sequentially unrelated classes; cyclophilins, FKBPs and Parvullins [17]. Moonlighting functions of prolyl isomerases include virulence character [18], stress response [19,20], cell cycle regulation [21,22], chromatin remodeling [23], transcription factor regulation [24], RNA-mediated gene expression [25,26] etc. Infection biology is not only influenced by pathogen encoded Ppiases, but host Ppiases also play a crucial role in development of the disease. For example, human CypA and Cyp B bind to the capsid protein of HIV and facilitate internalization of the virion particles in CD4 cells. Additionally, human cyclophilin A (PpiA) is recruited with nascent HIV-1 virions as well as incoming HIV-1 capsids where it is involved in isomerization of an exposed proline [27].

*M*.*tb* has two cyclophilins, PpiA and PpiB coded by *ppiA* (Rv0009) and *ppiB* (Rv2582) respectively, which are located apart in the genome. *M*.*tb* PpiA is known to be structurally and phylogenetically related to eukaryotic cyclophilins. It has been previously reported that it is a secretory protein and interacts with several host proteins such as those involved in iron regulation, immune defense mechanism and signal transduction [28,29]. Presence of PpiB has been reported in proteomes of membrane fraction [30] and mannosylation enriched culture filtrate [31], which are indicative of its surface expression. PpiB is reported to be essential for the survival of the pathogen [32]. Functional characterization of the enzymes reflecting their possible role as a stress responder in the pathogen, and thus contributing to its virulence, has not been investigated so far. We describe the functional characterization of *M*.*tb* Ppiases (PpiA and PpiB) and demonstrate that they display chaperone-like activity. We show that recombinant *M*.*tb* Ppiases (PpiA and PpiB) expressed in *E*.*coli* could bind to heat labile MalZ protein *in-vitro* and can prevent its aggregation. *E*.*coli* transformants expressing *M*.*tb* Ppiases exhibited increased survival under heat shock, as compared to vector control. That these cyclophilins enabled the survival of HEK293T cells under conditions of hypoxic and oxidative stress pointed to the potential role of *M*.*tb* Ppiases *in vivo* to absorb cellular stress.

## Materials and Methods

### Materials

IPTG, imidazole, N-succinyl- Ala-Ala-Pro-Phe-p-nitroanilide, trifluoroethanol, LiCl, α-chemotrypsin, 8-anilino-1-naphthalene-sulfonic acid (ANS), reduced Glutathione, DTT and MTT were obtained from Sigma. All cell culture reagents were obtained from GIBCO. All enzymes were purchased from NEB (USA); ELISA kit from Peprotech and toxicity removal kit from Norgen. All reagents used were analytical grade. The plasmids and strains used in this study are listed (S1 Supporting Information).

### Enzyme assay of purified recombinant Ppiases

The ORF encoding *M*.*tb ppiA* (Rv0009) and *M*.*tb ppiB* (Rv2582) were PCR amplified from *M*.*tb* H_37_Rv genomic DNA by using forward and reverse primers. *ppiA* was cloned in pET28a vector using *Bam*HI and *Hin*dIII restriction sites and *ppiB* in pGEX6p1 vector using *Bam*H1 and *Xho*1 restriction sites. Recombinant proteins were purified using Ni-NTA column for PpiA and glutathione sepharose affinity column for PpiB as described earlier [33].Endotoxin removal was achieved by passing the recombinant protein through polymyxin B resins as described [34].PPIase activity of both, rPpiA and rPpiB was evaluated using a spectrophotometric assay [35]. 8mM oligo peptide N-succinyl-Ala-Ala-Pro-Phe-p-nitroanilide substrate solution was prepared in trifluoroethanol containing 0.45 M LiCl in cold. Coupled enzyme α-Chymotrypsin was prepared at a concentration of 60mg/ml in cold solubilizing buffer (33 μl of 1 mM HCl and 2mM CaCl_2_ solution). Reaction mixture included 910μl 0.1 M TrisCl, pH 8.0, 50μl 600μM α-Chymotrypsin and 30μl rPpiA which were incubated for 2 minutes at 15^°^C. Subsequent addition of 10μl of peptide solution resulting in a final concentration of 80μM initiated the reaction. The enzyme catalyzed cis–trans isomerization of Ala-Pro bond, coupled with cleavage of the trans peptide by α-chymotrypsin was observed as increase in absorbance at 390nm at 15^°^C. Measurements were recorded every 0.5 sec till 3 minutes and the final absorbance was measured from each curve. The absorbance at each time point was subtracted from that value.

### ANS Fluorescence of rPpiases

Fluorescence of ANS in the presence of rPpiA and rPpiB was measured by exciting at 390 nm and following the emission between 450 and 550 nm [36]. 0.5mg/ml of the recombinant proteins, rPpiA and rPpiB, respectively was incubated with 50 μM ANS for 30 min at room temperature, and fluorescence of protein-bound dye was recorded. Fluorescence emission spectrum of ANS alone was used as control. The spectra were corrected with appropriate buffer and protein blanks. The emission and excitation slit widths were set at 10 and 10nm, respectively.

### Aggregation Assay

Gel filtration chromatography was carried out to know the oligomeric states of rPpiA and rPpiB in solution and the protein profile was compared with protein molecular size standards.Further to know the presence of hydrophobic surfaces which are associated with chaperon activity, 3D model of PpiB was constructed by submitting amino acid sequence to SWISS-MODEL[37]. Crystal structure of PpiA was downloaded from protein data bank (PDB ID:1W74). PyMOL program was used to carry out Molecular visualization and general analysis [38].Chaperone activity of rPpiases was investigated in terms of its ability to prevent aggregation of MalZ. MalZ loses its native conformation and undergoes aggregation during incubation at 47°C. MalZ and GroEL were purified for the assay as reported earlier [39]. Assay for MalZ aggregation was performed in presence and absence of rPpiA or rPpiB at 47°C. Light scattering was measured by recording the absorbance as described earlier [40]. The samples used for the assay involved (0.4uM) MalZ alone, rPpiA alone, rPpiA with lysozyme (negative control) and purified GroEL (positive control) and with increasing molar ratios of rPpiA (10, 20, 40) and rPpiB (5, 10, 20).

### Residual activity of denatured *Nde*1

*Nde*1 (10 U) was incubated at 60°C for 20 min in the absence or presence of rPpiases (rPpiA and rPpiB). BSA (20 μg) was used as a control [36] Assessment of the residual enzyme activity was assayed by digesting 150ng of circular pUC18 at 37°C for 1 h. The digestion mixture was electrophoresed on 1% agarose gel, stained with ethidium bromide and visualized under UV light in a Gel doc system (Bio-Rad).

### Growth rescue of *E*. *coli* from thermal shock

Rescue of *E*. *coli* cells from thermal shock was performed using the method reported previously [36]. Fold survival with and without heat shock was calculated and the value was normalized taking the vector control (pET28A and pGEX6p1) as one fold.

### Cloning of *ppiA* and *ppiB* for expression in HEK 293T cells

Standard procedures were followed for cloning. The genes coding for *ppiA* and *ppiB* were amplified using *M*. *tuberculosis* H_37_Rv genomic DNA as template. For PCR amplification*ppiA* forward primer with *Xho*I site (GCCCTCTAGACTCGAGATGGCAGACTGTGATTCC) and reverse primer with *Hin*dIII site (GTTTAAACTTAAGCTTGGAGATGGTGATCGACTCGA) and similarly for *ppiB* forward primer with *Xho*I site (CCCTCTAGACTCGAGATGGGCCACTTGACACCG) and reverse primer with *Hin*dIII site (GTTTAAACTTAAGCTTATCCAGCAGCACCGACGTGA) were used. The PCR was carried out as described [41] and PCR products and pcDNA3.1mychis (-) vector were digested and ligation reactions were set up. Recombinant plasmid constructs were transfected into the HEK293T cells (obtained from NCCS, Pune, India) by lipofection.

### Survival assays under various stresses by MTT assay

It has been reported that H_2_O_2_ and CoCl_2_ induce oxidative stress and hypoxia, respectively in mammalian cells [42–45].Assessment of the hypoxia stress and oxidative stress on the proliferation of HEK293T cells was carried out by MTT assay as described [46]. Cells were transfected with pcDNA_ppiA, pcDNA_ppiB and vector alone. 3 million cells were taken in 35 mm dish in each category. For hypoxia, 5000 cells/well from each category were seeded in a 96-well plate cultured for 12 hours at 37°C in 5% CO_2_ in 150ul complete RPMI1640. Dose dependent treatment with CoCl_2_ (50, 100, 150, 200 μM) was performed for 24 hours [47]. Untransfected cells, only media control and empty wells were used as controls. At the end of the treatment, MTT 25μl (from 5mg/ml in PBS), was added and then incubated for 4 hours. Acidic isopropanol (4%HCl and 0.1% NonidetP-40) was added in each well after removal of the supernatant. After shaking the plate for 10 min, cell viability was assessed by measuring the absorbance at 590 nm with 620 reference filter. Similarly, resistance to oxidative stress was determined by treating the cells expressing PpiA and PpiB, respectively with 10–40μM H_2_O_2_ in increasing concentrations_._ All assays were carried out in triplicate.

## Results

### Recombinant Ppiases are enzymatically active

**Fig 1:**
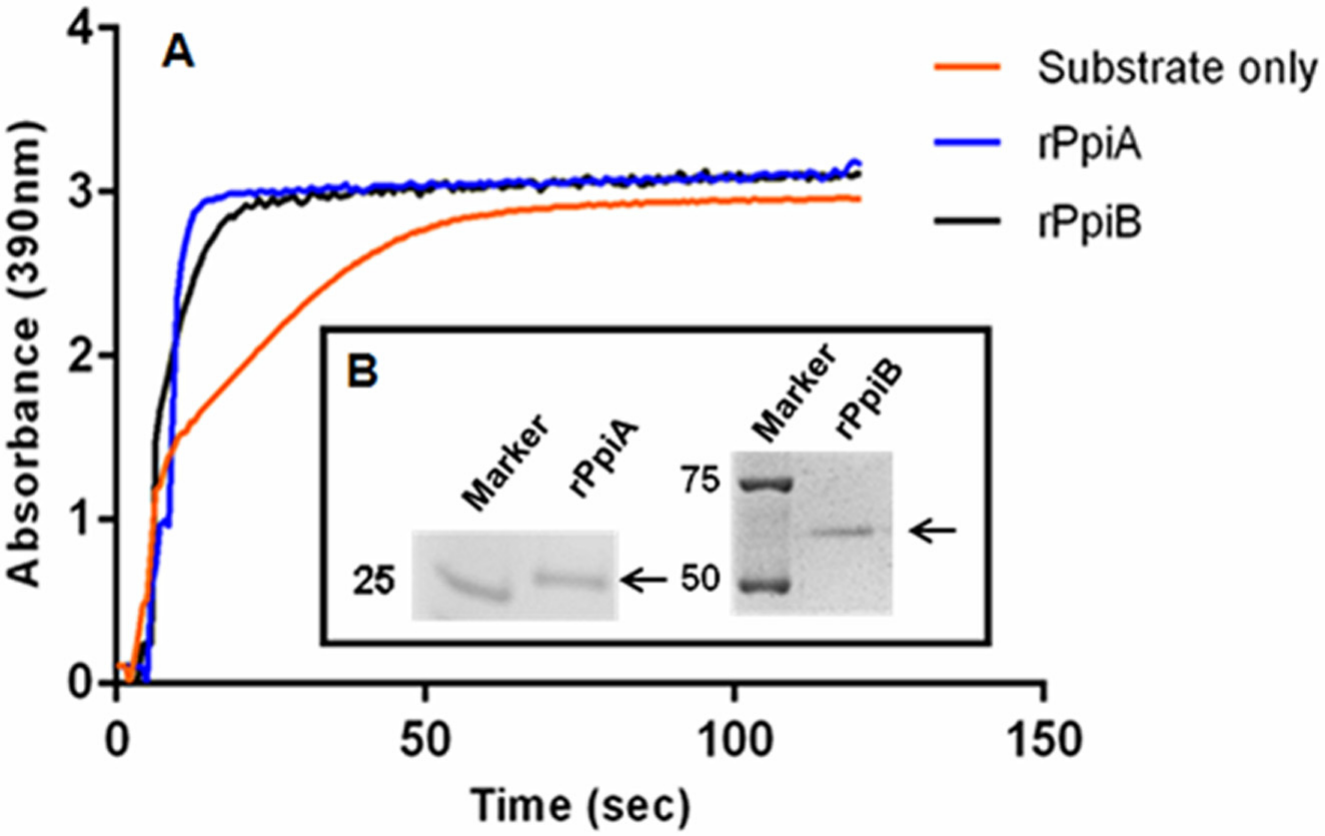
Purified rPpiases of *M*.*tb* are enzymatically active. **A.** Isomerization activity of rPpiA and rPpiB at a concentration of 50nM was measured in a coupled assay using the chromogenic peptide N-succinyl-Ala-Ala-Pro-Phe-p-nitroanilide and α chymotrypsin compared with the spontaneous background rate of cis-trans isomerization in the absence of the recombinant enzymes. **B.** Molecular weight of purified histidine-tagged rPpiA and GST tagged rPpiB as checked on 10% SDS polyacrylamide gel was around 25kDa and 69kDa, respectively.

His tagged *M*.*tb* rPpiA and GST tagged rPpiB was purified using Ni-NTA column and glutathione sepharose affinity column, respectively. PpiB was less stable in the absence of GST due to its structural complexity. We tried to remove GST, but in the absence of GST tag PpiB got precipitated. rPpiA displayed the expected 25kDa molecular size while rPpiB protein band was observed at 69kDA molecular size after SDS gel. Enzymatic activity of the purified proteins was measured in a spectrophotometric-coupled assay using the chromogenic peptide N-succinyl-Ala-Ala-Pro-Phe-p-nitro-anilide and α chymotrypsin at 15^°^C (Fig 1). Increase in the rate of isomerization activity as compared to the control show that the recombinant Ppiases are enzymatically active [28].

### *M*.*tb* Ppiases display chaperone like activity as evident by surface hydrophobicity

**Fig 2:**
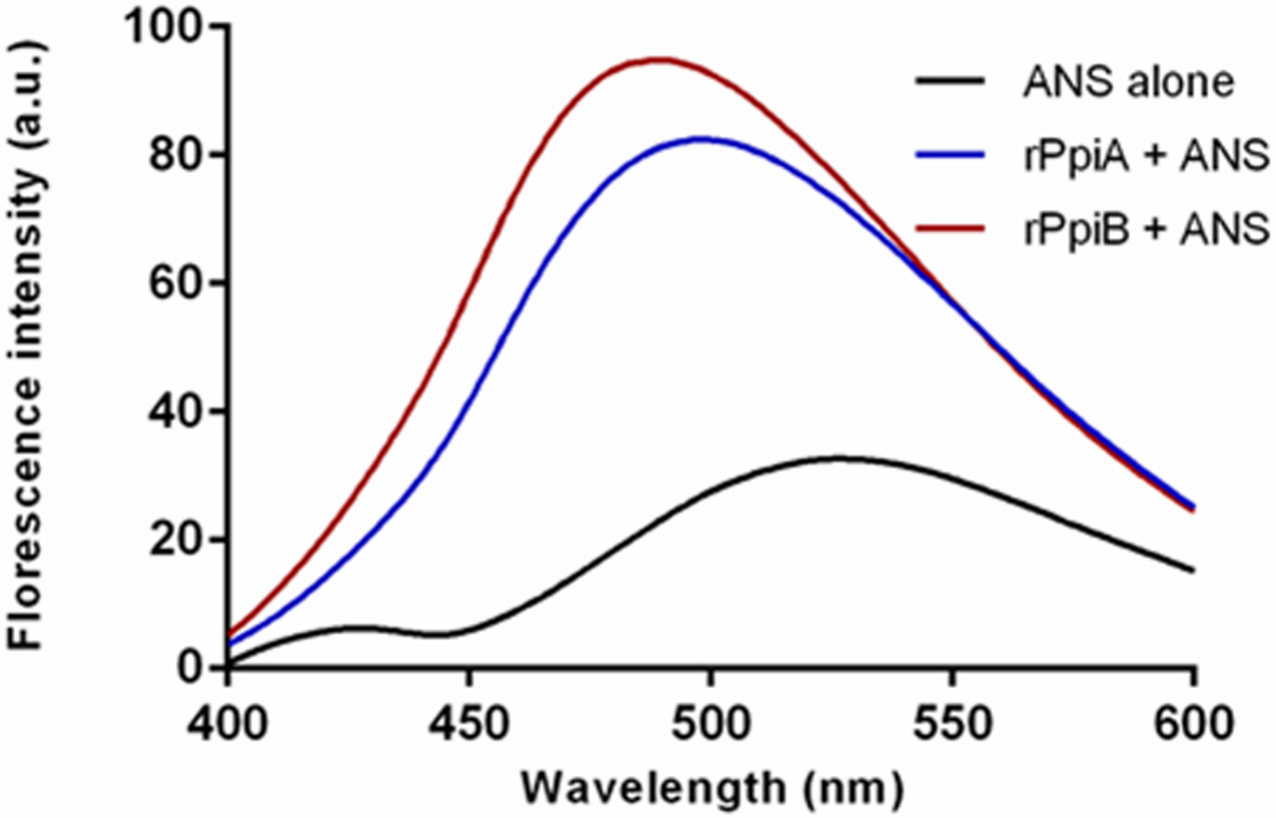
ANS Florescence spectra reveal surface hydrophobicity in *M*.*tb* rPpiases. Concentration of ANS, rPpiA and rPpiB used were 20μM, 0.1 mg/ml and 0.1mg/ml, respectively. Blue shift in the position of peak and increase in the intensity of peak was observed upon addition of rPpiA and rPpiB. The ANS emission was scanned in the range of 400 to 600 nm.

**Fig 3:**
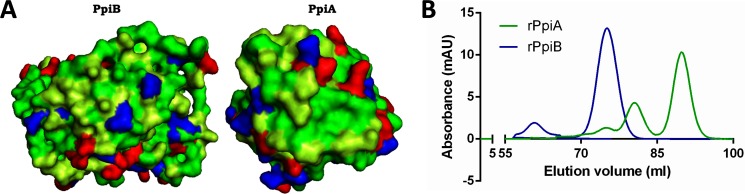
PpiA and PpiB have hydrophobic patches on the surface and are present in oligomeric form in solution. **A.** Green color shows the hydrophobic patches on the surface of protein structures of PpiA and PpiB, blue, red and limon yellow colors show negative, positive and polar residues, respectively. **B.** Elution profile of Gel Filtration Chromatography: Green color indicates elution profile of rPpiA and blue indicates elution profile of rPpiB.

ANS has been commonly used as a fluorescent probe to establish surface hydrophobicity in proteins. A blue shift of fluorescence emission maxima and increase of fluorescence intensity is generally attributed to the hydrophobicity of a binding site [48].The relative fluorescence intensity and maximum emission wavelength of ANS alone and ANS bound to rPpiA and rPpiB was measured by exciting at 390nm. The maximum emission wavelength of ANS alone was found to be 540nm and a clear blue shift in the emission wavelength was observed in case of ANS bound to rPpiA and rPpiB (Fig 2). A significant increase in the fluorescent intensity could be noticed when rPpiA and rPpiB was bound to ANS. These results confirmed surface hydrophobicity in PpiA and PpiB of *M*.*tb*. which is known to be associated with chaperone like function [36]. We constructed 3D model of PpiB and compared it with PpiA that confirms the presence of hydrophobic patches on the surface of these proteins (Fig 3A). The ANS fluorescence spectra and presence of hydrophobic patches on the surface of PpiA and PpiB clearly point to the likely function of *M*.*tb* Ppiases as a chaperone.

### rPpiases protect MalZ from thermal aggregation

**Fig 4:**
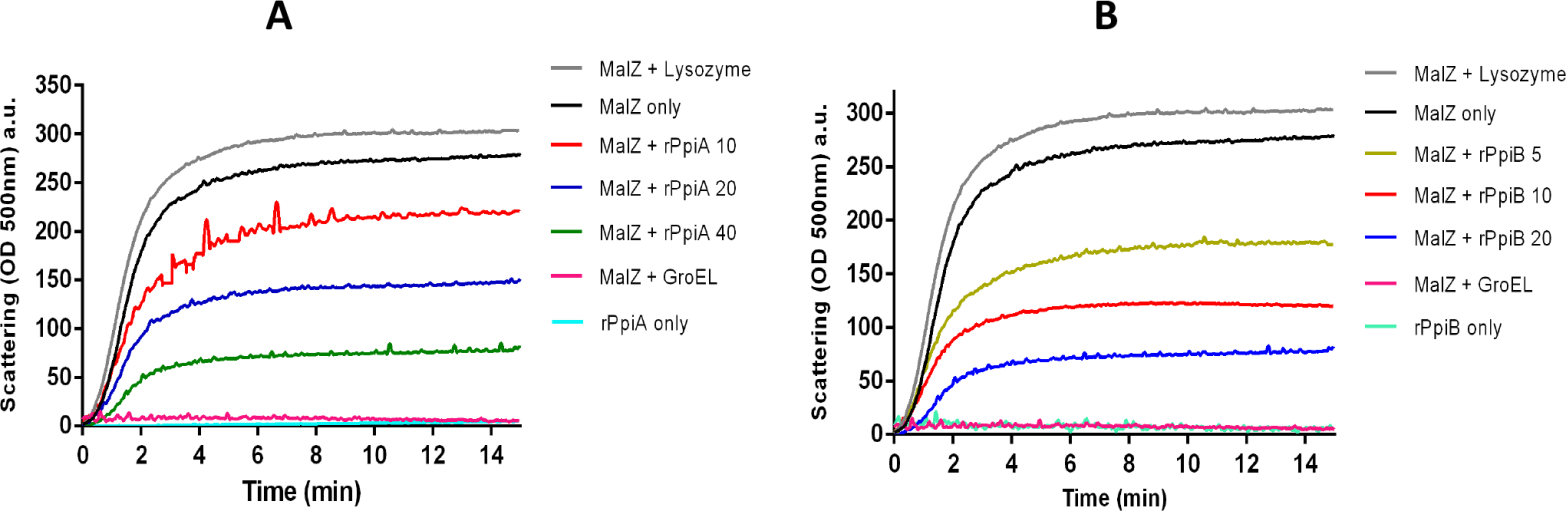
*M*.*tb* rPpiases suppress aggregation of MalZ. The aggregation pattern was monitored by light scattering at O.D. 500 nm with excitation and emission slit width 5 and 2.5 nm, respectively. GroEL was used as a positive control. Lysozyme was used as a negative control. **A.** Increasing molar ratio of rPpiA (10, 20, 40) was used. **B**. Increasing molar ratio of rPpiB (5,10, 20) was used.

The elution profile of the recombinant proteins confirmed the presence of oligomeric states of rPpiA and rPpiB in solution, as oligomeric rearrangement in solution is one of the characteristic of chaperon like proteins Fig 3B).To experimentally demonstrate chaperone like activity of *M*.*tb* rPpiases, their ability to prevent thermal aggregation of a heterologous protein MalZ was assessed. MalZ is known to aggregate under elevated temperature[39]. Light scattering assay to monitor aggregation was carried out by assessing absorbance (500nm) of thermally denatured MalZ in presence and absence of rPpiA and rPpiB. The thermal stability of rPpiases was also monitored and as expected for chaperones, both the rPpiases were highly stable at 47^°^C, exhibiting negligible aggregation. When rPpiA was co-incubated with MalZ in increasing molar ratio (10, 20, 40), it was able to increasingly prevent aggregation at 47^°^C (Fig 4A). In comparison to rPpiA, rPpiB could inhibit aggregation of MalZ at almost half the concentration (Fig 4B). The use of appropriate positive (GroEL) and negative control (lysozyme) confirmed the specificity of the aggregation inhibition activity by the rPpiases. Molecular chaperones are known to exhibit thermal stability and can protect proteins from thermal denaturation and aggregation [36] and these result therefore, clearly demonstrate that *M*.*tb* peptidyl prolyl isomerases can protect proteins from thermal aggregation directly pointing to their chaperone like activity.

### *M*.*tb* rPpiases protect *Nde*1 from thermal denaturation and consequent loss of restriction enzyme activity

**Fig 5:**
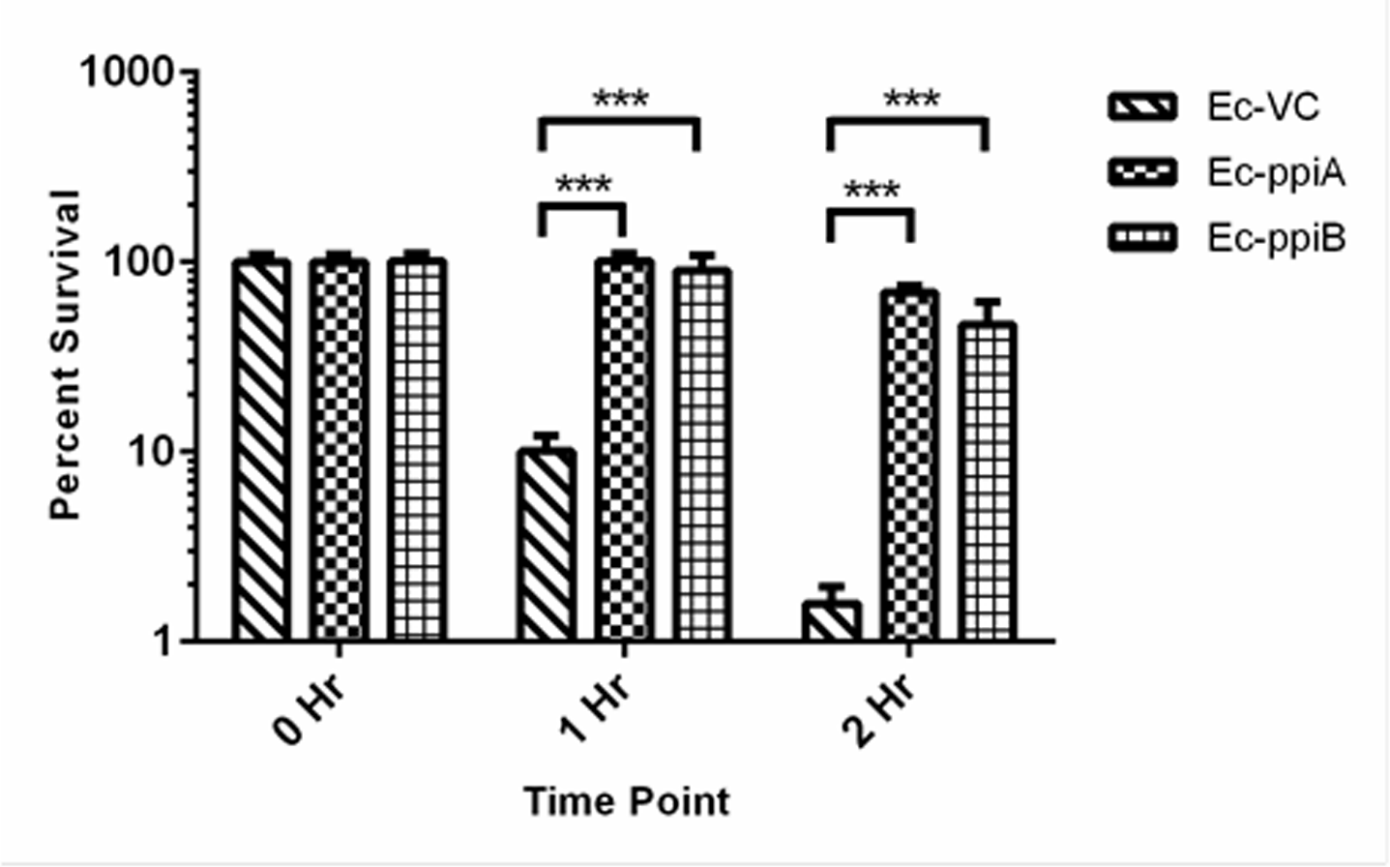
rPpiases can rescue *E*.*coli* from thermal shock: *E*.*coli* expression strain was transformed with Ec_VCy, pGEX6p-1 only, Ec_ppiA and Ec_ppiB. After heat treatment at 50^°^C different dilutions of 100ul culture was plated at one hour interval. The quantification of bacterial growth was carried out by counting the bacterial colony forming unit (cfu).*E*. *coli* transformed with *ppiA* and *ppiB* exhibited approximately 10 fold more survival compared with vector control.

Having shown that rPpiases could protect MalZ from thermal aggregation, we further investigated the ability of rPpiases to protect the enzymatic activity of *Nde*1 restriction enzyme from thermal denaturation. Plasmid pET22b has a single restriction enzyme site for the enzyme *Nde*1. Upon heat denaturation *Nde*1 loses its ability to linearize pET22b plasmid (Fig 5, lane 3), but when heat denatured in the presence of rPpiA (lane 4) or rPpiB (lane 5) it retained the ability to digest and could linearize the pET22B plasmid DNA. BSA when used as a control could not protect *Nde*1 and as a consequence the enzyme activity was lost after heat denaturation (lane 6). These results further demonstrate that *M*.*tb* rPpiases cannot only protect proteins from thermal aggregation but can also preserve the functional activity of proteins under *in vitro* conditions.

### rPpiases can rescue *E*. *coli* from thermal shock

**Fig 6:**
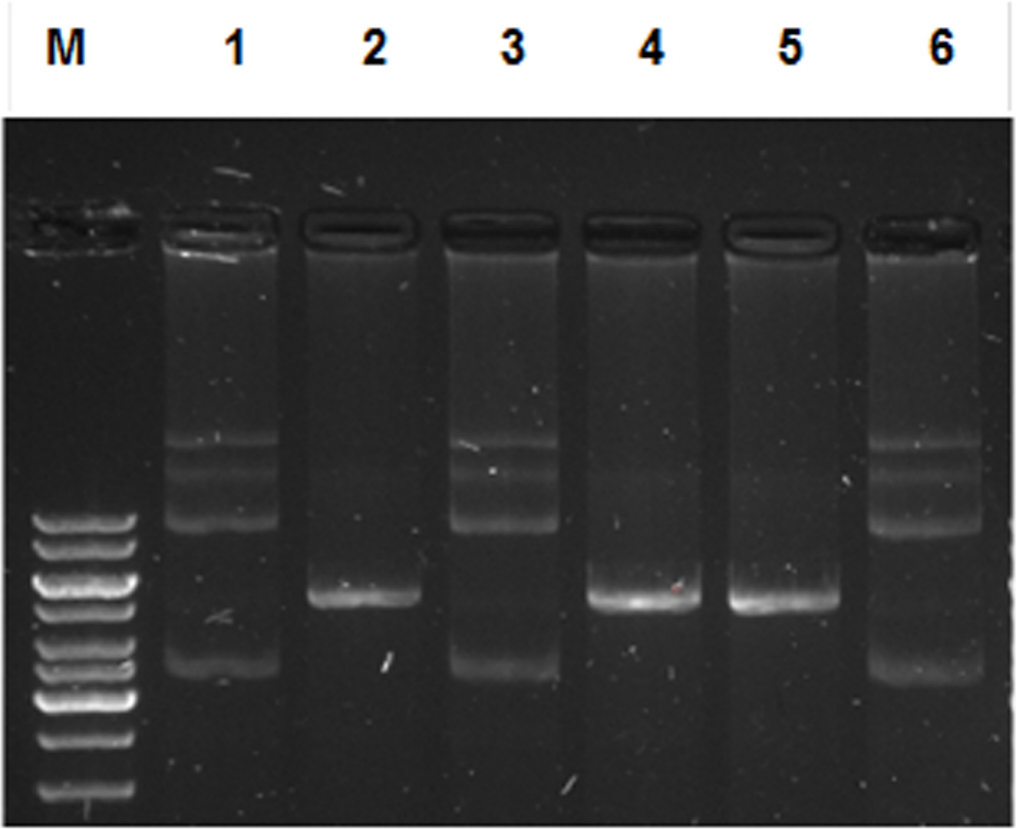
rPpiases protected *Nde*1 is refractile to thermal denaturation. Enzymatic activity of thermal denatured *Nde*1 was assayed in presence and absence of rPpiA, rPpiB and control protein BSA. Lane M, 1-kb molecular size marker; lane 1, uncut pET22b; lane 2, pET22b digested with native *Nde*1; lane 3, with heat denatured *Nde*1; lane4, with rPpiA treated heat denatured *Nde*1; lane 5, with rPpiB treated heat denatured *Nde*1; lane 6, with BSA treated heat denatured *Nde*1.

In order to assay for chaperone like function of rPpiases under physiological conditions, by checking if *E*.*coli* expressing *M*.*tb* PpiA and PpiB is resistant to thermal shock as compared to vector control. *E*.*coli* transformants were assessed for their ability to grow after thermal shock (50^°^C) as described earlier [36]. Results indicate that *E*. *coli* cells transformed with *M*.*tb ppiA* and *ppiB* showed more than ten folds survival after one hour and about 80 fold survival after 2 hours as compared to the *E*.*coli* transformed with vector alone (Fig 6). These results provide conclusive evidence that rPpiases show chaperone like function both under in *in-vitro* conditions and also under physiological conditions.

### *M*.*tb* Ppiases confer protection to HEK293T cells against oxidative stress and hypoxia

**Fig 7:**
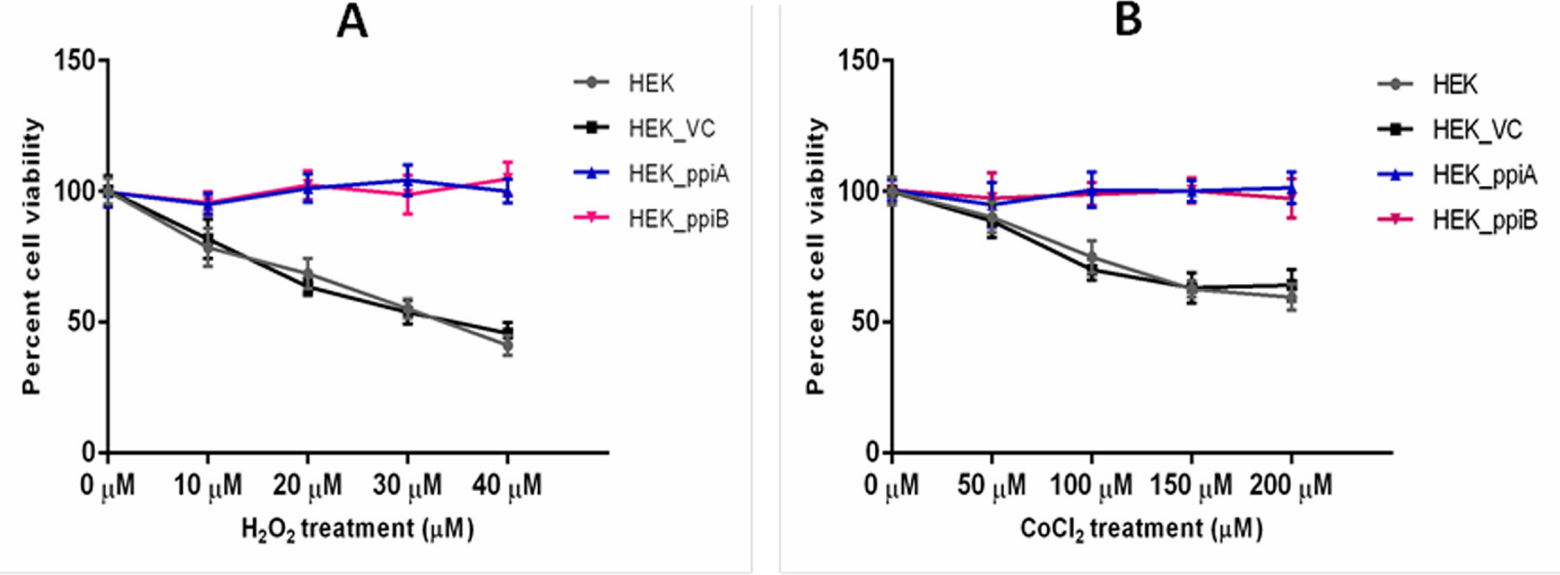
*M*.*tb* Ppiases impart resistance to HEK293T cells against hypoxic and oxidative stress. MTT assay was performed to score the percent cell viability of HEK293t cells transiently expressing PpiA and PpiB under oxidative stress (panel **A)** or hypoxia (panel **B**) induced by treatment of cells with varying concentrations of H_2_O_2_ (0, 10, 20, 30 and 40μM) or CoCl_2_ (0, 50, 100, 150 and 200μM), respectively. The data are representative of three independent experiments. Error bar represents mean ± S.D.

Results presented so far demonstrated the ability of *M*.*tb* Ppiases not only to act as a chaperone, under *in-vitro* conditions and but also under physiological conditions. Given the fact that chaperones help in maintaining cellular homeostasis under various stress conditions [49] and their presence increases tolerance to heat, toxins, hypoxic shock and increase cell longevity by maintaining proteostasis, we designed experiments to investigate whether expression of Ppiases in HEK cells cultured *in-vitro* impart resistance against hypoxia and oxidative stress. For this MTT assay was performed to determine viability of HEK cells expressing PpiA and PpiB proteins after oxidative stress and hypoxia. HEK cells transfected with vector alone was used as a control. For oxidative stress cells were treated with different concentration of H_2_O_2_ (0, 10, 20, 30, 40 μM) whereas for hypoxic stress the cells were treated with varying concentrations of CoCl_2_ (0, 50, 100, 150, 200 μM). A significant increase in viability of cells expressing PpiA and PpiB (Fig 7), as compared to vector control, could be seen. These observations clearly implicate *M*.*tb* Ppiases, in aiding the intracellular survival of the pathogen amid the hostile environment of infected cells pointing to its likely role *in-vivo*.

## Discussion

A multifaceted interplay between the host immune response and pathogen virulence factors govern the outcome of the infection caused by *Mycobacterium tuberculosis*. Peptidyl-prolyl isomerase A of *M*.*tb*, also known as cyclophilin A is secreted in intraphagosomal niche and is known to be upregulated during infection [50]. Other than ubiquitous expression, prolyl isomerases have large number of isoforms and are localized in different subcellular locations in higher organisms, e.g., in *Saccharomyces*, 8 different prolyl isomerases are targeted to endoplasmic reticulum, mitochondria and nucleus from cytosol [51]. Isoform diversity and various subcellular localization are indicative of their functional importance. Recently, Ppiases have been studied in different contexts such as cell regulatory processes [52], role in different cancer status [53,54] and inflammation [55] and other abnormalities [56, 57]. In addition to their vitality in protein folding, peptidyl prolyl isomerases have also been implicated in many pathological conditions like diabetes, asthma, cancer and microbial infections. *surA* gene coding for Ppiase is associated with virulence of pathogenic strains of *Salmonella*, *E*. *coli* and *Helicobacter pylori*. Mutation in *surA* gene significantly affects virulence potential of these pathogens [58,59].

In the present study, we observed that *M*.*tb* Ppiases exhibit chaperone-like activity. Surface hydrophobicity is considered important during the interaction of molecular chaperones with misfolded proteins [60,61]. ANS binding analysis of rPpiases showed increase in absorbance and blue shift in the emission maxima, a reflection of its chaperone-like activity. Unlike Mal Z, *M*.*tb* rPpiases were refractile to thermal aggregation however; when MalZ was incubated along with rPpiA and rPpiB at 45^°^C aggregation was inhibited. About 90% of the enzymatic activity of thermally denatured *Nde*1was restored when incubated with rPpiA and rPpiB. Molecular chaperones can transiently bind and stabilize an unstable conformation of a protein by preventing its misfolding and aggregation.With *in-vitro* evidence of rPpiases as a probable chaperone, we further investigated its chaperone like function *in-vivo*. It was clearly evident that survival of *E*.*coli* transformed with *ppiA* and *ppiB* of *M*.*tb* was quantitatively higher compared to the vector control. Similar examples of proteins that showed chaperone-like activity have been reported earlier [36, 62–64].

The ability of *M*.*tb* to grow under reduced oxygen conditions and resist oxidative stress is directly correlated to its ability to cause disease. Although O_2_is essentially needed for survival of *M*.*tb*, it can easily adapt itself to the hypoxic microenvironment of tissue lesions, sites of active TB and inside microphages [65]. In the Wayne model [66], under gradual hypoxic growth environment, bacteria move away from proliferative cycle and tend towards the latent form that is adapted to hypoxia and remain viable for extended period. Turning to hypoxia tolerant status appears to be the key response mechanism for coping stress, and other survival challenges [12]. In the present study, we sought to investigate the role of *M*.*tb* Ppiases in conferring protection to HEK293T cells under hypoxia and oxidative stress. A significantdifference in the survival of HEK cells transiently expressing PpiA and PpiB proteins as compared to the untransformed cells was evident. There are several other mycobacterial HSPs which could impart survival amid hostile host effector functions such as hypoxia and oxidative stress [67]. The induction of proinflammatory response by the human macrophages upon infection with *M*.*tb* is a natural defense strategy mounted by the host. Infection itself induces cellular stress resulting in an unfolded protein response (UPR). Human resistin—a pro-inflammatory cytokine [68], along with other chaperones, is over expressed in response to UPR and aids in protein folding [36]. *M*.*tb* once lodged within the macrophages may induce similar stress resulting in misfolding of mycobacterial proteins, conditions that may lead to molecular crowding detrimental for the bacteria. To combat such strategies mounted by the host, *M*.*tb* deploys its Ppiases to assist in folding of the misfolded proteins. Therefore, it is very likely that these Ppiases act as a connecting link between infection, inflammation, stress response and protein misfolding, thereby assisting in the survival of the *M*.*tb*within the hostile environment of the macrophages. In conclusion, our data demonstrating chaperone-like function of *Mycobacterium tuberculosis* Ppiases have implications in virulence and pathogenicity terms of possible role inenhancing the stress tolerance of the pathogen.

## Supporting Information

S1 Supporting Information Strains and plasmids used in this study. (DOCX)

## References

[1] WHO TB Report. Global tuberculosis report. 2015.

[2] Ehrt S, Schnappinger D. Mycobacterial survival strategies in the phagosome: defence against host stresses. Cell Microbiol. 2009;11: 1170–1178. 10.1111/j.1462-5822.2009.01335.x 19438516 PMC3170014

[3] Russell DG. Mycobacterium tuberculosis: here today, and here tomorrow. Nat Rev Mol Cell Biol. Nature Publishing Group; 2001;2: 569–77. 10.1038/35085034 11483990

[4] Henderson B. Prokaryotic and Eukaryotic Heat Shock Proteins in Infectious Disease Pockley AG, Calderwood SK, Santoro MG, editors. Proteins. Dordrecht: Springer Netherlands; 2010;4: 185–209. 10.1007/978-90-481-2976-8

[5] Ng VH, Cox JS, Sousa AO, MacMicking JD, McKinney JD. Role of KatG catalase-peroxidase in mycobacterial pathogenisis: Countering the phagocyte oxidative burst. Mol Microbiol. 2004;52: 1291–1302. 10.1111/j.1365-2958.2004.04078.x 15165233

[6] Cirillo SLG, Subbian S, Chen B, Weisbrod TR, Jacobs WR, Cirillo JD. Protection of Mycobacterium tuberculosis from reactive oxygen species conferred by the mel2 locus impacts persistence and dissemination. Infect Immun. 2009;77: 2557–67. 10.1128/IAI.01481-08 19349422 PMC2687327

[7] Mukhopadhyay S, Nair S, Hasnain SE. Nitric oxide: Friendly rivalry in tuberculosis. Curr Signal Transduct Ther. Bentham Science Publishers; 2007;2: 121–128. 10.2174/157436207780619536

[8] Divangahi M, Chen M, Gan H, Desjardins D, Hickman TT, Lee DM, et al Mycobacterium tuberculosis evades macrophage defenses by inhibiting plasma membrane repair. Nat Immunol. 2009;10: 899–906. 10.1038/ni.1758 19561612 PMC2730354

[9] Shukla S, Richardson ET, Athman JJ, Shi L, Wearsch PA, McDonald D, et al Mycobacterium tuberculosis lipoprotein LprG binds lipoarabinomannan and determines its cell envelope localization to control phagolysosomal fusion. PLoS Pathog. 2014;10: e1004471 10.1371/journal.ppat.1004471 25356793 PMC4214796

[10] Huang D, Bao L. Mycobacterium tuberculosis EspB protein suppresses interferon-γ-induced autophagy in murine macrophages. J Microbiol Immunol Infect. 2014; 10.1016/j.jmii.2014.11.008 25641595

[11] Kugelberg E. Immune evasion: Mycobacteria hide from TLRs. Nat Rev Immunol. Nature Publishing Group, a division of Macmillan Publishers Limited. All Rights Reserved.; 2014;14: 62–3. 10.1038/nri3604 24378842

[12] Gupta A, Kaul A, Tsolaki AG, Kishore U, Bhakta S. Mycobacterium tuberculosis: immune evasion, latency and reactivation. Immunobiology. 2012;217: 363–74. 10.1016/j.imbio.2011.07.008 21813205

[13] Tundup S, Mohareer K, Hasnain SE. Mycobacterium tuberculosis PE25/PPE41 protein complex induces necrosis in macrophages: Role in virulence and disease reactivation? FEBS Open Bio. 2014;4: 822–8. 10.1016/j.fob.2014.09.001 25379378 PMC4219985

[14] Stebbins CE. Structural microbiology at the pathogen-host interface. Cell Microbiol. 2005;7: 1227–36. 10.1111/j.1462-5822.2005.00564.x 16098211

[15] Wang P, Heitman J. The cyclophilins. Genome Biol. 2005;6: 226 10.1186/gb-2005-6-7-226 15998457 PMC1175980

[16] Galat A. Peptidylproline cis-trans-isomerases: immunophilins. Eur J Biochem. 1993;216: 689–707. 8404888 10.1111/j.1432-1033.1993.tb18189.x

[17] Kromina K a., Ignatov a. N, Abdeeva I a. Role of peptidyl-prolyl-cis/trans-isomerases in pathologic processes. Biochem Suppl Ser. 2008;2: 195–202. 10.1134/S199074780803001X

[18] Bell A, Monaghan P, Page AP. Peptidyl-prolyl cis-trans isomerases (immunophilins) and their roles in parasite biochemistry, host-parasite interaction and antiparasitic drug action. Int J Parasitol. 2006;36: 261–76. 10.1016/j.ijpara.2005.11.003 16443228

[19] Sykes K, Gething MJ, Sambrook J. Proline isomerases function during heat shock. Proc Natl Acad Sci U S A. 1993;90: 5853–7. 7685914 10.1073/pnas.90.12.5853 PMC46821

[20] Mark PJ, Ward BK, Kumar P, Lahooti H, Minchin RF, Ratajczak T. Human cyclophilin 40 is a heat shock protein that exhibits altered intracellular localization following heat shock. Cell Stress Chaperones. 2001;6: 59–70. 11525244 10.1379/1466-1268(2001)006<0059:hciahs>2.0.co;2 PMC434384

[21] Arevalo-Rodriguez M, Heitman J. Cyclophilin A is localized to the nucleus and controls meiosis in Saccharomyces cerevisiae. Eukaryot Cell. 2005;4: 17–29. 10.1128/EC.4.1.17-29.2005 15643056 PMC544151

[22] Lu KP, Hanes SD, Hunter T. A human peptidyl-prolyl isomerase essential for regulation of mitosis. Nature. 1996;380: 544–7. 10.1038/380544a0 8606777

[23] Wu X, Wilcox CB, Devasahayam G, Hackett RL, Arévalo-Rodríguez M, Cardenas ME, et al The Ess1 prolyl isomerase is linked to chromatin remodeling complexes and the general transcription machinery. EMBO J. 2000;19: 3727–38. 10.1093/emboj/19.14.3727 10899126 PMC313980

[24] Yang WM, Yao YL, Seto E. The FK506-binding protein 25 functionally associates with histone deacetylases and with transcription factor YY1. EMBO J. 2001;20: 4814–25. 10.1093/emboj/20.17.4814 11532945 PMC125595

[25] Thapar R. Roles of Prolyl Isomerases in RNA-Mediated Gene Expression. Biomolecules. 2015;5: 974–99. 10.3390/biom5020974 25992900 PMC4496705

[26] Krishnan N, Titus MA, Thapar R. The prolyl isomerase pin1 regulates mRNA levels of genes with short half-lives by targeting specific RNA binding proteins. PLoS One. Public Library of Science; 2014;9: e85427 10.1371/journal.pone.0085427 PMC3887067 24416409

[27] Keckesova Z, Ylinen LMJ, Towers GJ. Cyclophilin A Renders Human Immunodeficiency Virus Type 1 Sensitive to Old World Monkey but Not Human TRIM5 α Antiviral Activity. J Virol. 2006;80: 4683–4690. 10.1128/JVI.80.10.4683 16641261 PMC1472055

[28] Henriksson LM, Johansson P, Unge T, Mowbray SL. X-ray structure of peptidyl-prolyl cis-trans isomerase A from Mycobacterium tuberculosis. Eur J Biochem. 2004;271: 4107–13. 10.1111/j.1432-1033.2004.04348.x 15479239

[29] Bhaduri A, Misra R, Maji A, Bhetaria PJ, Mishra S, Arora G, et al Mycobacterium tuberculosis cyclophilin A uses novel signal sequence for secretion and mimics eukaryotic cyclophilins for interaction with host protein repertoire. PLoS One. 2014;9: e88090 10.1371/journal.pone.0088090 24505389 PMC3913756

[30] Gu S, Chen J, Dobos KM, Bradbury EM, Belisle JT, Chen X. Comprehensive proteomic profiling of the membrane constituents of a Mycobacterium tuberculosis strain. Mol Cell proteomicsMCP. 2003;2: 1284–96. 10.1074/mcp.M300060-MCP200 14532352

[31] Cole ST, Brosch R, Parkhill J, Garnier T, Churcher C, Harris D, et al Deciphering the biology of Mycobacterium tuberculosis from the complete genome sequence. Nature. Nature Publishing Group; 1998;393: 537–544. 9634230 10.1038/31159

[32] Sassetti CM, Boyd DH, Rubin EJ. Genes required for mycobacterial growth defined by high density mutagenesis. Mol Microbiol. 2003;48: 77–84. 12657046 10.1046/j.1365-2958.2003.03425.x

[33] Banerjee S, Nandyala AK, Raviprasad P, Ahmed N, Hasnain SE. Iron-dependent RNA-binding activity of Mycobacterium tuberculosis aconitase. J Bacteriol. 2007;189: 4046–52. 10.1128/JB.00026-07 17384188 PMC1913386

[34] Banerjee S, Nandyala A, Podili R, Katoch VM, Murthy KJR, Hasnain SE. Mycobacterium tuberculosis (Mtb) isocitrate dehydrogenases show strong B cell response and distinguish vaccinated controls from TB patients. Proc Natl Acad Sci U S A. 2004;101: 12652–7. 10.1073/pnas.0404347101 15314217 PMC514659

[35] Fischer G, Bang H, Mech C. Determination of enzymatic catalysis for the cis-trans-isomerization of peptide binding in proline-containing peptides. Biomed Biochim Acta. 1984;43: 1101–11. 6395866

[36] Suragani M, Aadinarayana VD, Pinjari AB, Tanneeru K, Guruprasad L, Banerjee S, et al Human resistin, a proinflammatory cytokine, shows chaperone-like activity. Proc Natl Acad Sci U S A. 2013;110: 20467–72. 10.1073/pnas.1306145110 24282299 PMC3870731

[37] Arnold K, Bordoli L, Kopp J, Schwede T. The swiss-model workspace: A web-based environment for protein structure homology modelling. Bioinformatics. 2006;22(2):195–201 16301204 10.1093/bioinformatics/bti770

[38] Delano WL. 2002 The PyMOL molecular graphics system Delano Scientific, San Carlos, CA http://www.pymol.org/.

[39] Paul S, Chaudhuri TK. Chaperone mediated solubilization of 69-kDa recombinant maltodextrin glucosidase in Escherichia coli. J Appl Microbiol. 2008;104: 35–41. 10.1111/j.1365-2672.2007.03519.x 18171380

[40] Goyal M, Chaudhuri TK, Kuwajima K. Irreversible denaturation of maltodextrin glucosidase studied by differential scanning calorimetry, circular dichroism, and turbidity measurements. PLoS One. Public Library of Science; 2014;9: e115877 10.1371/journal.pone.0115877 25548918 PMC4280130

[41] Bhuwan M, Arora N, Sharma A, Khubaib M, Pandey S, Chaudhuri TK, et al Interaction of Mycobacterium tuberculosis virulence factor RipA with MoxR1, a chaperone, is required for transport through TAT secretion sustem. mBio; 2016. In press. 10.1128/mBio.02259-15 PMC4810496 26933057

[42] Wijeratne SS, Cuppett SL, Schlegel V. Hydrogen peroxide induced oxidative stress damage and antioxidant enzyme response in Caco-2 human colon cells. J Agric Food Chem. 2005;53(22):8768–74. 16248583 10.1021/jf0512003

[43] Nakamura J, Purvis ER, Swenberg JA. Micromolar concentrations of hydrogen peroxide induce oxidative DNA lesions more efficiently than millimolar concentrations in mammalian cells. Nucleic Acids Res. 2003;31(6):1790–5. 12626721 10.1093/nar/gkg263 PMC152865

[44] Triantafyllou A, Liakos P, Tsakalof A, Georgatsou E, Simos G, Bonanou S. Cobalt induces hypoxia-inducible factor-1alpha (HIF-1alpha) in HeLa cells by an iron-independent, but ROS-, PI-3K- and MAPK-dependent mechanism. Free Radic Res. 2006;40(8):847–56. 17015263 10.1080/10715760600730810

[45] Piret JP, Mottet D, Raes M, Michiels C. CoCl2, a chemical inducer of hypoxia-inducible factor-1, and hypoxia reduce apoptotic cell death in hepatoma cell line HepG2. Ann N Y Acad Sci. 2002;973:443–7. 12485908 10.1111/j.1749-6632.2002.tb04680.x

[46] Khan N, Rahim SS, Boddupalli CS, Ghousunnissa S, Padma S, Pathak N, et al Hydrogen peroxide inhibits IL-12 p40 induction in macrophages by inhibiting c-rel translocation to the nucleus through activation of calmodulin protein. Blood. American Society of Hematology; 2006;107: 1513–20. 10.1182/blood-2005-04-1707 16249388

[47] Piret J-P, Mottet D, Raes M, Michiels C. CoCl2, a chemical inducer of hypoxia-inducible factor-1, and hypoxia reduce apoptotic cell death in hepatoma cell line HepG2. Ann N Y Acad Sci. 2002;973: 443–7. 12485908 10.1111/j.1749-6632.2002.tb04680.x

[48] Gasymov OK, Glasgow BJ. ANS fluorescence: potential to augment the identification of the external binding sites of proteins. Biochim Biophys Acta. 2007;1774: 403–11. 10.1016/j.bbapap.2007.01.002 17321809 PMC2039916

[49] Henderson B, Martin A. Bacterial virulence in the moonlight: Multitasking bacterial moonlighting proteins are virulence determinants in infectious disease. Infect Immun. 2011;79: 3476–3491. 10.1128/IAI.00179-11 21646455 PMC3165470

[50] Målen H, Berven FS, Fladmark KE, Wiker HG. Comprehensive analysis of exported proteins from Mycobacterium tuberculosis H37Rv. Proteomics. 2007;7: 1702–18. 10.1002/pmic.200600853 17443846

[51] Arevalo-Rodriguez M, Wu X, Hanes SD, Heitman J, others. Prolyl isomerases in yeast. Front Biosci. 2004;9: 2420–2446. 15353296 10.2741/1405

[52] Lin C-H, Li H-Y, Lee Y-C, Calkins MJ, Lee K-H, Yang C-N, et al Landscape of Pin1 in the cell cycle. Exp Biol Med. 2015;240: 403–8. 10.1177/1535370215570829 PMC4935233 25662955

[53] Yao Q, Li M, Yang H, Chai H, Fisher W, Chen C. Roles of cyclophilins in cancers and other organ systems. World J Surg. 2005;29: 276–80. 10.1007/s00268-004-7812-7 15706440

[54] Chen J, Chen S, Wang J, Zhang M, Gong Z, Wei Y, et al Cyclophilin J is a novel peptidyl-prolyl isomerase and target for repressing the growth of hepatocellular carcinoma. PLoS One. 2015;10: e0127668 10.1371/journal.pone.0127668 26020957 PMC4447340

[55] Liu T, Schneider RA, Lee NY, Hoyt DG. Peptidylprolyl cis/trans isomerase, NIMA-interacting 1 (PIN1) regulates pulmonary effects of endotoxin and tumor necrosis factor-α in mice. Biochem Biophys Res Commun. 2014;452: 468–72. 10.1016/j.bbrc.2014.08.089 25159840

[56] Tsurubuchi T, Allender E V, Siddiqui MR, Shim K-W, Ichi S, Boshnjaku V, et al A critical role of noggin in developing folate-nonresponsive NTD in Fkbp8 -/- embryos. Child’s Nerv Syst. 2014;30: 1343–53. 10.1007/s00381-014-2428-1 24817375

[57] Restelli M, Lopardo T, Lo Iacono N, Garaffo G, Conte D, Rustighi A, et al DLX5, FGF8 and the Pin1 isomerase control ΔNp63α protein stability during limb development: a regulatory loop at the basis of the SHFM and EEC congenital malformations. Hum Mol Genet. 2014;23: 3830–42. 10.1093/hmg/ddu096 24569166 PMC4065156

[58] Sydenham M, Douce G, Bowe F, Ahmed S, Chatfield S, Dougan G. Salmonella enterica serovar typhimurium surA mutants are attenuated and effective live oral vaccines. Infect Immun. 2000;68: 1109–15. 10678914 10.1128/iai.68.3.1109-1115.2000 PMC97255

[59] Basak C, Pathak SK, Bhattacharyya A, Pathak S, Basu J, Kundu M. The secreted peptidyl prolyl cis,trans-isomerase HP0175 of Helicobacter pylori induces apoptosis of gastric epithelial cells in a TLR4- and apoptosis signal-regulating kinase 1-dependent manner. J Immunol (Baltimore, Md1950). 2005;174: 5672–80. 10.4049/jimmunol.174.9.5672 15843568

[60] Das KP, Surewicz WK. Temperature-induced exposure of hydrophobic surfaces and its effect on the chaperone activity of alpha-crystallin. FEBS Lett. 1995;369: 321–5. 7649280 10.1016/0014-5793(95)00775-5

[61] Yang H, Huang S, Dai H, Gong Y, Zheng C, Chang Z. The Mycobacterium tuberculosis small heat shock protein Hsp16.3 exposes hydrophobic surfaces at mild conditions: conformational flexibility and molecular chaperone activity. Protein Sci. 1999;8: 174–179. 10.1110/ps.8.1.174 10210195 PMC2144111

[62] Freeman BC, Toft DO, Morimoto RI. Molecular chaperone machines: chaperone activities of the cyclophilin Cyp-40 and the steroid aporeceptor-associated protein p23. Science (80-). 1996;274: 1718–1720. 10.1126/science.274.5293.1718 8939864

[63] Ou WB, Luo W, Park YD, Zhou HM. Chaperone-like activity of peptidyl-prolyl cis-trans isomerase during creatine kinase refolding. Protein Sci. 2001;10: 2346–53. 10.1110/ps.23301 11604540 PMC2374073

[64] Lilie H, Lang K, Rudolph R, Buchner J. Prolyl isomerases catalyze antibody folding in vitro. Protein Sci. 1993;2: 1490–1496. 10.1002/pro.5560020913 8104614 PMC2142458

[65] Boon C, Dick T. Mycobacterium bovis BCG Response Regulator Essential for Hypoxic Dormancy. J Bacteriol. 2002;184: 6760–6767. 10.1128/JB.184.24.6760 12446625 PMC135468

[66] Wayne LG. Dormancy of Mycobacterium tuberculosis and latency of disease. Eur J Clin Microbiol Infect Dis. 1994;13: 908–14. 7698116 10.1007/BF02111491

[67] Yuan Y, Crane DD, Simpson RM, Zhu YQ, Hickey MJ, Sherman DR, et al The 16-kDa alpha-crystallin (Acr) protein of Mycobacterium tuberculosis is required for growth in macrophages. Proc Natl Acad Sci U S A. 1998;95: 9578–83. 9689123 10.1073/pnas.95.16.9578 PMC21381

[68] Silswal N, Singh AK, Aruna B, Mukhopadhyay S, Ghosh S, Ehtesham NZ. Human resistin stimulates the pro-inflammatory cytokines TNF-α and IL-12 in macrophages by NF-κB-dependent pathway. Biochem Biophys Res Commun. 2005;334: 1092–1101. 10.1016/j.bbrc.2005.06.202 16039994

