# Supplementary material for: Mycobacterium tuberculosis Peptidyl-Prolyl Isomerases Also Exhibit Chaperone like Activity In-Vitro and In-Vivo
Source: PLoS One. 2016 Mar 16;11(3):e0150288. doi: 10.1371/journal.pone.0150288 (PMC4794191; doi:10.1371/journal.pone.0150288)
Supplement: S1 Supporting Information — (DOCX) [file pone.0150288.s001.docx]

**TABLE S1. Strains and plasmids used in this study**

**Plasmids Relevant characteristics Source/Reference**

pET28α Kan^R^ pET28a (cloning vector), pBR322 origin Novagen

pET28_ppiA pET28a containing *ppiA* gene with T7 promoter This work

pGEX_ppiB pGEX6p1 containing *ppiB* gene with T7 promoter This work

pcDNA 3.1 (-) Mammalian expressing vector, Neomycin^R^, myc & his tag, Invitrogen

CMV promoter

pcDNA_ppiA pcDNA3.1(-) containing *ppiA* gene This work

pcDNA_ppiB pcDNA3.1(-) containing *ppiB* gene This work

**Strains Relevant characteristics Source/Reference**

DH5α *supE44* *ΔlacU(Φ80lacZΔM*1*5) hsdR*1*7 rec*1 *endA*1 Novagen

*gyrA96 thi-*1 *relA*1

BL-21(DE3) *F– ompT gal dcm lon hsdSB(rB- mB-) λ(DE3 [lacI lacUV5-* Novagen

*T7 gene* 1*ind*1 *sam7 nin5])*

Ec_ppiA BL-21 λ (DE3) containing pET28a-ppiA This work

Ec_ppiB BL-21 λ (DE3) containing pGEX-ppiB This work

Selection marker resistant to Ampicillin (*amp^R^*) and kanamycin (*kan^R^*)
